# Circular RNA circMYL1 Inhibit Proliferation and Promote Differentiation of Myoblasts by Sponging miR-2400

**DOI:** 10.3390/cells10010176

**Published:** 2021-01-16

**Authors:** Ibrahim Elsaeid Elnour, Xiaogang Wang, Toremurat Zhansaya, Zhanerke Akhatayeva, Rajwali Khan, Jie Cheng, Yongzhen Hung, Xianyong Lan, Chuzhao Lei, Hong Chen

**Affiliations:** 1 Key Laboratory of Animal Genetics, Breeding and Reproduction of Shaanxi Province, College of Animal Science and Technology, Northwest A&F University, Yangling 712100, China; ibrahim@nwafu.edu.cn (I.E.E.); dg20350012@smail.nju.edu.cn (X.W.); toremuratzhansaya@nwafu.edu.cn (T.Z.); akhatayevazhanerke@nwafu.edu.cn (Z.A.); rajwalikhan@nwafu.edu.cn (R.K.); chengjie1212@nwafu.edu.cn (J.C.); hyzsci@nwafu.edu.cn (Y.H.); lanxianyong79@nwsuaf.edu.cn (X.L.); leichuzhao1118@nwafu.edu.cn (C.L.); 2 Faculty of Veterinary Science, University of Nyala, Nyala 155, Sudan

**Keywords:** circMYL1, miR2400, bovine primary myoblasts, proliferation, differentiation

## Abstract

Circular RNAs (circRNAs) are a class of endogenous non-coding RNAs (ncRNAs) involved in regulating skeletal muscle development by sponging miRNAs. In this study, we found that the circMYL1 expression was down-regulated during myoblast proliferation, while gradually up-regulated in myoblast differentiation. The potential role of circMYL1 was identified in the proliferation of bovine myoblast through mRNA and protein expression of proliferation marker genes (*PCNA*, *CyclinD1*, and *CDK2*), cell counting kit-8 assay, flow cytometry analysis, and 5-ethynyl 2′-deoxyuridine (EdU) assay. Analysis of the expression of differentiation marker genes (*MyoD*, *MyoG*, and *MYH2*) and immunofluorescence of Myosin heavy chain (MyHC) was used to assess cell differentiation. The proliferation analysis revealed that circMYL1 inhibited the proliferation of bovine primary myoblast. Furthermore, the differentiation analysis demonstrated that circMYL1 promoted the differentiation of bovine primary myoblast. The luciferase screening and RNA immunoprecipitation (RIP) assays found that circMYL1 could have interaction with miR-2400. Additionally, we demonstrated that miR-2400 promoted proliferation and inhibited differentiation of bovine primary myoblast, while circMYL1 may eliminate the effects of miR-2400, as showed by rescue experiments. Together, our results revealed that a novel circular RNA of circMYL1 could inhibit proliferation and promote differentiation of myoblast by sponging miR-2400.

## 1. Introduction

Mammals have three different types of muscle tissues, including cardiac muscle, skeletal muscle, and smooth muscle [1]. Skeletal muscle represents half of the total body mass and has a critical role for voluntary motion and support [1]. The vertebrate skeletal muscle originates from the somites during embryonic development [2]. Previous studies showed that myogenesis is a complicated and orchestrated process, regulated by several genes, signalling pathways, and network regulation [3,4]. For instance, the multiple specifics myogenic regulatory transcription factors (MRFs) are important for the regulation of skeletal muscle development. Previous studies found that MRFs are a member of the basic helix-loop-helix (bHLH) family, including MYOG, MyoD, Myf5, and MRF4. MRFs that significantly expressed in skeletal muscle suggested that they have an essential role during the proliferation and differentiation of skeletal muscle [5]. In addition, non-coding RNAs such as microRNAs (miRNAs), long non-coding RNAs (lincRNAs) and circular RNAs (circRNAs) have been reported to be involved in the development of skeletal muscle and play critical roles during myogenesis [6,7,8]. Nevertheless, to explore the molecular regulatory mechanisms during muscle development and meat quality need further in-depth experiments [9].

Circular RNAs (circRNAs) are a class of endogenous non-coding RNAs (ncRNAs) that have a covalently closed circular structure with no 5′ end cap and 3′ end poly(A) tail, more stable compared with linear RNA molecules, produced by back-splicing from precursor mRNA [10,11]. circRNAs have high expression in the cytoplasm of eukaryotic cells [12,13], and can be circRNAs can exist for a long time in various cell and tissue at specific development stages [14]. Recent studies have divided the circRNAs into three groups; (1) exon-derived circRNAs (exonic circRNAs), which are formed by exon splicing of the coding gene, including one or Multiple exon sequences [15,16]; (2) Intron circRNA (circular intronic RNA, ciRNA), which without of exon sequences and produced by cyclization of the coding gene intron sequences [17]; (3) exon-intron circRNA (EIciRNA), which is a fusion circRNA that contains both an exon and an intron sequence [18,19]. Furthermore, it is found that circRNAs are involved in the transcription and post-transcriptional regulation of gene through different modes of action [18]. Previous studies noted that most circRNAs contain miRNA binding sites. Therefore, circRNA can act as a highly efficient competitive endogenous RNA (ceRNA), effectively sponging miRNAs and regulating miRNA target genes [20,21]. For instance, circRNAs, ciRS-7, and Sry inhibit the function of miRNA through sponging miR-7 and miR-138, respectively [13,22]. Besides sponging miRNAs, circular RNA may also bind to RNA-binding proteins (RBPs) to form complexes of RNA proteins (RPCs) [23]. In addition, it was stated that (EIciRNAs) interact with small nuclear ribonucleoproteins of RNA polymerase II and U1 and cis-induce host-gene transcript in the nucleus [24]. cirRNAs have been reported to play critical roles during skeletal muscle proliferation and differentiation. For instance, it was noted that the circFGFR4 overexpression promotes myogenic differentiation of bovine primary myoblasts by sponging miR-107 [25]. Furthermore, it was also investigated that the circZfp609 sponges miR-194-5p to inhibit myogenic differentiation in C2C12 [26]. Other research has also found that circLMO7 overexpression promotes proliferation and inhibits bovine primary myoblasts differentiation by sponging miR-378a-3p [27]. Similarly, CircFUT10 inhibits proliferation and promotes differentiation of bovine primary myoblasts by sponging miR-133a [28], whereas the overexpression of circSNX29 promotes myoblasts differentiation and inhibits myoblast proliferation by sponging miR-744 and activates the Wnt5a/Ca^2+^ signalling pathway [29]. We stated in our previous research that circMYL1 expressed differently between adult and fetal of bovine muscle, suggesting that circMYL1 may affect the development of muscles [27].

MicroRNAs (miRNAs) are about 17 to 24 nucleotides in length and are a class of small single-stranded endogenous, non-coding RNAs. Usually, they bind to specific target mRNAs (3′-untranslated region (3′-UTR)) to induce mRNA degradation or translation inhibition [30]. Several studies reported that miRNA involved in multiple physiological and biochemical processes, such as carcinogenesis, hematopoiesis, neurogenesis, cell proliferation, and cell apoptosis [31,32]. Glazov et al. reported that miR-2400 is not recognizable in other mammalian genomes. Therefore, it is a specific bovine miRNA [33]. Recently, it was reported that the overexpression of miR-2400 promotes the proliferation of skeletal muscle-derived satellite cells (MDSCs) by targeting the *MYOG* gene [34]. However, the function of miR-2400 in the regulation of myoblast differentiation was not reported.

This study aimed to define the circMYL1 molecular mechanisms in the regulation of bovine myogenesis and to disclose its regulatory mechanism through miR-2400 interaction. We found that circMYL1 inhibits proliferation and promotes differentiation of bovine primary myoblasts by acting as a sponge of miR-2400 and activated MYOG gene. The findings may be fruitful to understand the role of circMYL1 in myogenesis and will provide a platform for the study of myogenesis based on circular RNA experiments.

## 2. Materials and Methods

### 2.1. Tissue Samples Collection

In this study, the animal samples have been approved by the Northwest Agriculture and Forestry University’s Animal Care and Use Committee. At a local slaughterhouse in (Xi’an; China), all specimens from Qinchuan bovine embryonic phase (90 days) were collected, including skeletal muscle, stomach, intestine, spleen, heart, liver, lung, and kidney, and washed by diethylpyrocarbonate (DEPC) water, and stored at −80 °C until RNA extraction.

### 2.2. RNA Extraction and Real-Time qPCR

Total cell or tissue RNAs were extracted using the manufacturer’s instructions for TRIzol Reagent (Takara, Dalian, China). Next, using the PrimeScript RT reagent kit with gDNA Eraser (Takara, Dalian, China), RNA was reversed to complementary DNA (cDNA). And a nucleoplasmic separation kit (PARIS kit, Life Technologies, Carlsbad, CA, USA) was used to separate the nuclear and cytoplasmic fractions. For treatment with RNase R, 1 μg of total RNA was incubated at 37 °C for 20 min with two units μg^−1^ of RNase R and then purified using an RNeasy MinElute cleaning kit (Qiagen, Hilden, Germany).The SYBR Green PCR master mix reagent kit (Takara, Dalian, China) was used to conduct RT-qPCR. GAPDH and U6 (for miRNA) were used as an internal control for data normalization with three biological replicates. The relative expression level of mRNA was calculated using the 2^−∆∆Ct^ technique. All primers used in this study are listed in Table S1.

### 2.3. Plasmid Construction

To explore the possible functions of circMYL1 during bovine skeletal muscle development, we downloaded the sequencing data of circRNAs of bovine muscle tissue from NCBI: accession ID, GSE87908; databank URL, https://www.ncbi.nlm.nih.gov/geo/query/acc.cgi?token=atglausktpsjlel&acc=GSE87908 (Supplementary Text S1). For the construction of circMYL1 overexpression plasmids, using PCR to amplify the linear sequences of circMYL1, and the cDNA template was produced from the bovine primary myoblasts RNA by RT-PCR. The obtained linear sequences were then cloned into the *KpnI* and *BamHI*-restriction sites of the pCD2.1 vector (Invitrogen, Carlsbad, CA, USA) according to the manufacturer’s instruction, to produce the overexpression vector PCD2.1-circMYL1. The MYOG 3′UTR-WT fragment, including the miR-2400 binding site, was amplified and inserted into the psiCHECK-2 vector (MYOG-WT) (Promega, Madison, WI, USA) at the 3‘ end of the Renilla gene using the *XhoI* and *NotI* restriction enzymes (Takara, Dalian, China) and T4 DNA ligase. Mutant type psiCHECK-2-MYOG 3′UTR-MUT (MYOG-MUT) was created using overlapping PCR to mutate complementary to the miR-2400 seed region. MYOG-WT and MYOG-MUT are eventually incorporated in the same way into the psiCHECK-2 vector. A miR-2400 sensor (with a perfect miR-2400 binding site) was created by inserting two sequences that complement the mature miR-2400 sequence after the Rluc psiCHECK-2 vector. Consequently, the fluorescence activity alteration will react to miR-2400 reflecting. The PCK-circMYL1 vector was also obtained using the same procedure. Primer sequences are shown in Table S2. All constructs were verified by sequencing (Sangon Biotech, Shanghai, China).

### 2.4. Cell Culture

The bovine primary myoblasts, as reported previously [35], were isolated and cultured from bovine longissimus muscle. The HEK293T cells (ATCC, Manassas, VA, USA) and bovine primary myoblasts were grown in growth medium containing high-glucose Dulbecco’s modified Eagle’s medium (DMEM; Hyclone, Marlborough, MA, USA) and supplemented by 10% or 20% fetal bovine serum (FBS) (Hyclone, Marlborough, MA, USA) and 1% penicillin-streptomycin (Invitrogen, Carlsbad, CA, USA) and incubated at 37 °C with 5% CO_2_. The differentiation of bovine primary myoblast was induced by replacing growth medium with the differentiation medium (DMEM containing 2% horse serum) when bovine primary myoblasts confluence reached approximately 70–80%.

### 2.5. Cell Transfection

The bovine primary myoblasts were cultured in six-well plates. When the confluence of the cells reached to 40% for proliferation or 70–80% for differentiation the cells were transfected with PCD2.1-circMYL1 (2 μg/mL), 100 nm si-circMYL1 (RiboBio, Guangzhou, China), 50 nm miR-2400 mimics (RiboBio, Guangzhou, China) using Lipofectamine 2000 (Invitrogen, Carlsbad, CA, USA) according to the manufacturer’s protocol. Transcription was inhibited by adding 1 mM of Actinomycin D solution to the cell culture medium (Leagene, Beijing, China).

### 2.6. RNA Fluorescent In Situ Hybridization (RNA-FISH)

Specific probes to circMYL1 sequence were used to perform in situ hybridization in bovine primary myoblasts following instructions from the probe manufacturer (RiboBio, Guangzhou, China). The probe sequence was 5′-GGCATTCATCTCTTCATAGTTGATGCA-3′. The cells were cultured in the six-well plates in cover glass and grown to a confluence of 70–80%, and then fixed. After treatment with 0.1% of Triton X-100 transmembrane, cells were incubated overnight at 37 °C with 20 mg/mL of circMYL1 probe. Then DAPI was used to counterstain the nuclei. Photos were obtained using a confocal laser microscope FV1200 (Olympus, Tokyo, Japan).

### 2.7. 5-Ethynyl 2′-Deoxyuridine Assay (EdU)

Cell-Light EdU Apollo^®^ (RiboBio, Guangzhou, China) was used to detect the proliferation of bovine primary myoblasts. The experiment was carried out following the protocols of the manufacturer. The bovine primary myoblast cells were grown in a 96-well plate and then transfected by treatments with three replicates in each group. Concisely, before immunostaining, the cells were incubated at 37 °C with 50 μm EdU for 2 h after 24 h of transfection. A fluorescent microscope (AMG EVOS, Seattle, DC, USA) was used to capture all images.

### 2.8. Cell Counting Kit-8 (CCK8) Assay

The cell counting reagent Kit-8 (CCK8) (Multiscience, Hangzhou, China) was used to assess the proliferation of cells. The bovine primary myoblasts were grown in 96-well plates with eight independent replicates for each treatment. After 24 h of cultured at 37 °C, we added 10 μL of CCK-8 reagent to each well for 4 h. Then an automatic microplate reader (Molecular Devices, San Jose, CA, USA) was used to measure the absorbance value of all samples at 450 nm.

### 2.9. Cell Cycle Assay by Flow Cytometry

Bovine primary myoblasts were seeded in 60 mm cell culture plates (2 × 10^6^ cells/well). After 24 h of transfected, we collected cells and washed with PBS buffer, and resuspended with 1 mL of DNA staining solution and 10 μL of permeabilization solution (Multisciences, Hangzhou, China). The suspension was then incubated for 30 min in the dark at room temperature. Flow Cytometry (FACS Canto TM II, BD Biosciences, San Jose, CA, USA) measured the cell cycle and, for each treatment group, there were three independent replicates.

### 2.10. Luciferase Activity Assay

For investigated the binding sites of circMYL1 with miR-2400, HEK293T cells were seeded in 96-well plates. Then, the miR-2400 mimics and PCK-circMYL1 or miR-2400 sensors and PCD2.1-circMYL1 were co-transfected with Lipofectamine 2000 into HEK293 T cells. The miR-2400 mimics and MYOG-WT or MYOG-MUT have also been co-transfected into cells. After 24 h, the cells were then washed by Phosphate Buffered Saline (PBS) and collected using 100 μL Passive Lysis Buffer (PLB). Luciferase activity assay was performed using a MPPC luminescence analyzer (HAMAMATSU, Beijing, China), and Dual-Luciferase^®^ Reporter (DLR) Assay Kit (Promega, Madison, WI, USA). The activities of Firefly luciferase were normalized in each well to Renilla luminescence.

### 2.11. Western Blotting Analysis

Total bovine primary myoblasts proteins were extracted from different treatment groups using RIPA lysis buffer containing 1mM PMSF (Solarbio; Beijing, China). The protein extract was then boiled for 10 min in a loading buffer of 5× SDS-PAGE. The protein was then separated by SDS-PAGE and transferred to a 0.2 μm polyvinylidene fluoride membranes (PVDF). Then incubated for 2 h at room temperature with 5% skim milk in Tris Buffered Saline, with Tween (TBST) to block the PVDF membrane. Subsequently, in 4 °C overnight incubation with the primary anti-MYOD specific antibodies (Dilution 1:1000; ab16148; Abcam, Cambridge, UK), anti-MYOG (1;1000; ab124800; Abcam), anti-MYH2 (Dilution 1:1000; WL02785; Wanleibio, Shenyang, China), anti-CDK2 (Dilution 1:1000; WL02028; Wanleibio), anti-PCNA (Dilution 1:500; WL03069; Wanleibio), anti-CyclinD1 (Dilution 1:1000; WL01435a; Wanleibio), anti-β-actin (1:1000; KM9001T, SungeneBiotech, Tianjin, China). After that, the PVDF membranes were washed with TBST buffer and then incubated at room temperature for 2 h with the secondary antibody. The secondary antibodies were: goat anti-mouse IgG HRP (M21001S, Abmart, Shanghai, China), goat anti-rabbit IgG (H + L)-HRP (BA1054, Boster, Wuhan, China). Finally, ECL luminous liquid (DiNing, Beijing, China) was used to detect the protein bands.

### 2.12. Immunofluorescent Staining

Primary bovine myoblasts seeded in 24-well plates and differentiation was induced to form the myotubes for four days. The differentiated cells were fixed for 30 min with 4% paraformaldehyde, then washed with PBS for 5 min three times. The cells were subsequently permeabilized by adding (Triton X-100) 0.5% for 15 min. The cells were then incubated overnight with the MyHC-antibody (1:250; GTX20015, GeneTex, Irvine, CA, USA), which was diluted in 1% Bovine Serum Albumin. The cells were washed with 1XPBS and incubated with IgG H+L secondary anti-mouse (Alexa Fluor 594) (1:500; RS3608, Immunoway, Plano, TX, USA) and protected for 2 h from light at room temperature. The nuclei of cells were stained for 15 min with DAPI (4′,6-diamidino-2-phenylindole) (Solarbio; Beijing, China). A fluorescence microscope was used to capture the Immunofluorescence images (DM5000B, Leica, Wetzlar, Germany).

### 2.13. Statistical Analysis

The differentiation index was calculated as the percentage of nuclei in MyHC-positive cells. The fusion index was calculated as the percentage of nuclei in fused myotubes out of the total nuclei. The data of the experiment are presented as mean ± SEM. SPSS 20.0 software was used for statistical analysis, and a Student’s *t*-test was used to determine the variation between the two groups. For multiple comparison analysis, data were analyzed by one-way ANOVA. Variations in * *p* < 0.05 and ** *p* < 0.01 were considered statistically significant.

## 3. Results

### 3.1. Characterization of circMYL1

**Figure 1:**
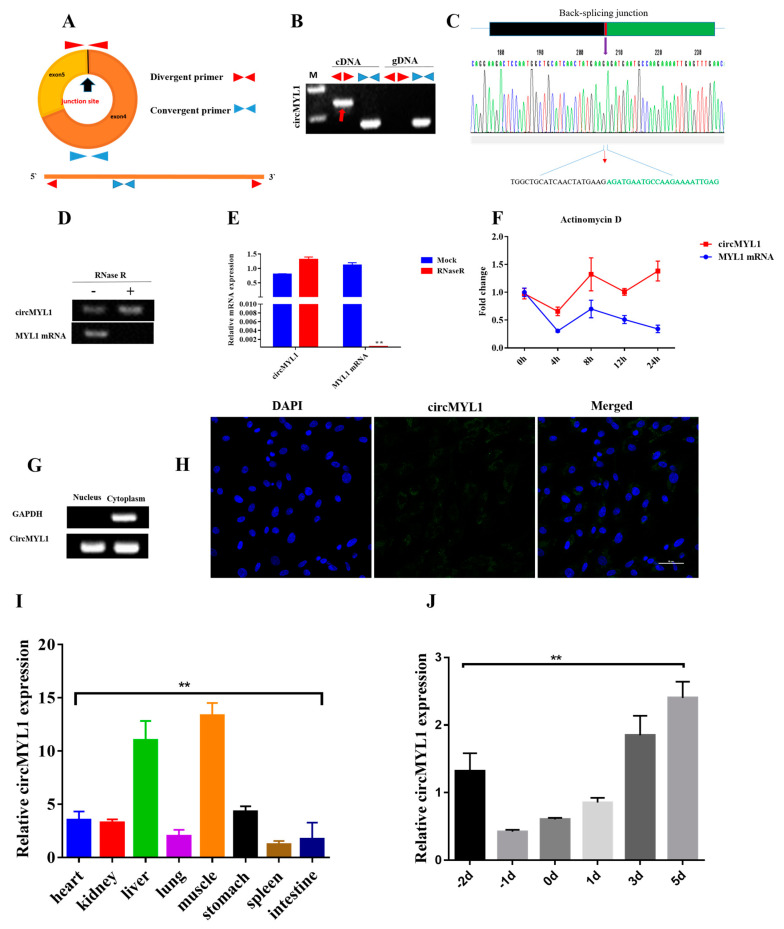
Characterization of circMYL1 (**A**) Schematic diagram showing the design of the circMYL1 primers. (**B**) The divergent primers and convergent primers in cDNA and gDNA samples. (**C**) Sanger sequencing confirmed the circMYL1 sequence of the back-splicing junction. (**D**) RNase R verified the existence of circMYL1. (**E**) qRT-PCR demonstrated the relative expression of circMYL1 and MYL1 mRNA in myoblasts treated with RNase R (**F**) qRT-PCR detected the relative expression of circMYL1and MYL1 mRNA in bovine primary myoblasts treated with Actinomycin D at the specific time points. (**G**) The cytoplasm and nuclear expression of circMYL1 were measured by semi-quantitative PCR. (**H**) RNA fluorescence in situ hybridization was performed to detect the subcellular location of circMYL1. Blue shows nuclei stained with DAPI, green shows the RNA probe detecting circMYL1. Scale bar, 50 μm. (**I**) The expression level of circMYL1 in different fetal tissues. (**J**) CircMYL1 expression level during proliferation and differentiation of bovine primary myoblasts. One-way ANOVA and *t*-tests were used for statistical analysis. Data are shown as mean ± SEM. *n* = 3. ** *p* < 0.01.

To confirm circMYL1′s circular nature, we designed two pairs of primers in a divergent and convergent direction (Figure 1A), the cDNA or genomic DNA (gDNA) was used as amplification templates. As shown in Figure 1B, the divergent primers produced only in cDNA samples a single distinct band; however, the convergent primers amplified the products from both the cDNA and the gDNA samples, which indicates that these circRNAs are bovine genome back-splicing products. The PCR products were sequenced using Sanger sequencing for the verification of the circMYL1 junction site (Figure 1C), which was consistent with the sequencing results. The circularity of circMYL1 was confirmed by RNase R. Total RNAs were treated with RNase R exonuclease, and qRT-PCR was performed, the results showed that the circMYL1 was more resistant to RNase R than MYL1 mRNA (Figure 1D,E). Furthermore, we investigated the stability and location of circMYL1 in bovine primary myoblast. After treatment with Actinomycin D, a transcription inhibitor, total RNA was collected at the specified time points. Then, we evaluated the expression level of circMYL1 and MYL1 mRNA. The results showed that the circMYL1 was highly stable with a half-life of transcript exceeding 24 h, while the corresponding linear transcript was the half-life of 4 h (Figure 1F). In order to investigate the cellular localization of circMYL1, we conducted a semi-quantitative PCR analysis of nuclear and cytoplasmic circMYL1 and RNA fluorescent in situ hybridization (FISH) with an RNA probe that precisely recognizes circMYL1′s back-splicing region to determine its subcellular location. Results showed that circMYL1 was located mainly in the cytoplasm and also was presented in the nucleus, indicating that circMYL1 could regulate gene expression at the post-transcription level and may regulate of their parental genes (Figure 1G,H). Moreover, we detected the expression level of circMYL1 in different developmental tissues using quantitative real-time polymerase chain reaction (qRT-PCR), to inspect the potential role of circMYL1 in growth and development, particularly in bovine muscle development (Figure 1I). The samples, including heart, liver, muscle, kidney, lung, spleen, stomach, and intestine, were collected from fetal cattle. The qRT-PCR analysis found that the circMYL1 showed a high expression level in some tissues, including muscle, liver, stomach, and heart, compared with intestine, kidney, lung, and spleen in the fetal stage. The circMYL1showed considerable expression pattern in muscle tissues, suggesting that circMYL1 plays an essential role in myogenesis. Furthermore, we detected the expression level of circMYL1during bovine primary myoblast proliferation and differentiation. We found that the expression level of circMYL1 gradually decreased during proliferation (3.21-fold) and increased from 0 d to 5 d during differentiation (4-fold) (Figure 1J). Taken together, our findings indicated that circMYL1 could be a positive regulator and stable circRNA for the development of muscles.

### 3.2. circMYL1 Inhibits Myoblasts Proliferation

**Figure 2:**
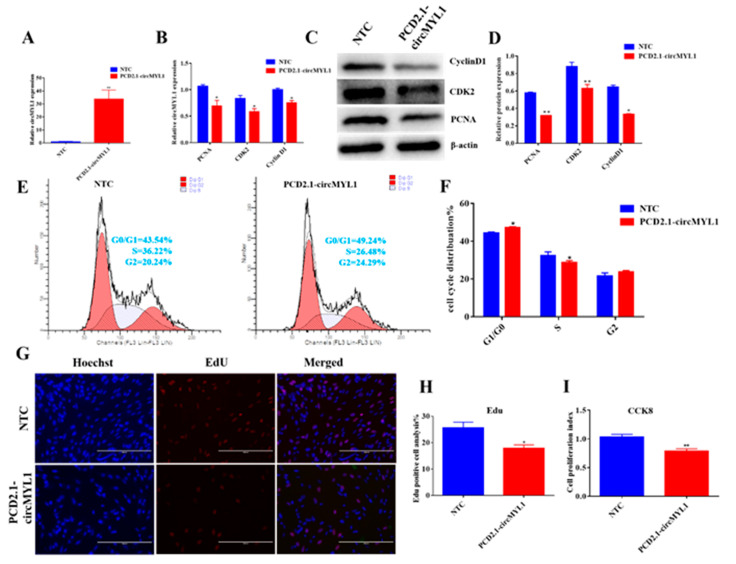
The overexpression of circMYL1 inhibits the proliferation of bovine primary myoblasts. (**A**) qRT-PCR detected the efficiency of the circMYL1 overexpression vector pCD2.1-circMYL1. (**B**) qRT-PCR detected the relative expression levels of proliferation marker genes *PCNA*, *CDK2*, and *CyclinD1*. (**C**) Western blot detected the protein expression levels of proliferation marker genes *PCNA*, *CDK2*, and *CyclinD1*. (**D**) Histogram graph showing the protein band density evaluated using ImageJ. (**E**) Bovine primary myoblasts were transfected with pCD2.1-circMYL1, and the cell cycle was analyzed by flow cytometry. (**F**) Histogram graph showing the number of cells in each phase of the cell cycle. (**G**) The cell proliferation was detected by 5-ethynyl 2′-deoxyuridine (EdU) assay after transfection of pCD2.1-circMYL1. EdU staining (red) for positive cells. DAPI staining (blue) for the cell nuclei. Scale bar, 200 μm. (**H**) Graph of a histogram showing the percentage of EdU-positive cells, and counted using ImageJ. (**I**) The cell counting kit-8 (CCK-8) assay was used to detect the cell proliferation index. Data are shown as mean ± SEM. *n* = 3. * *p* < 0.05; ** *p* < 0.01.

**Figure 3:**
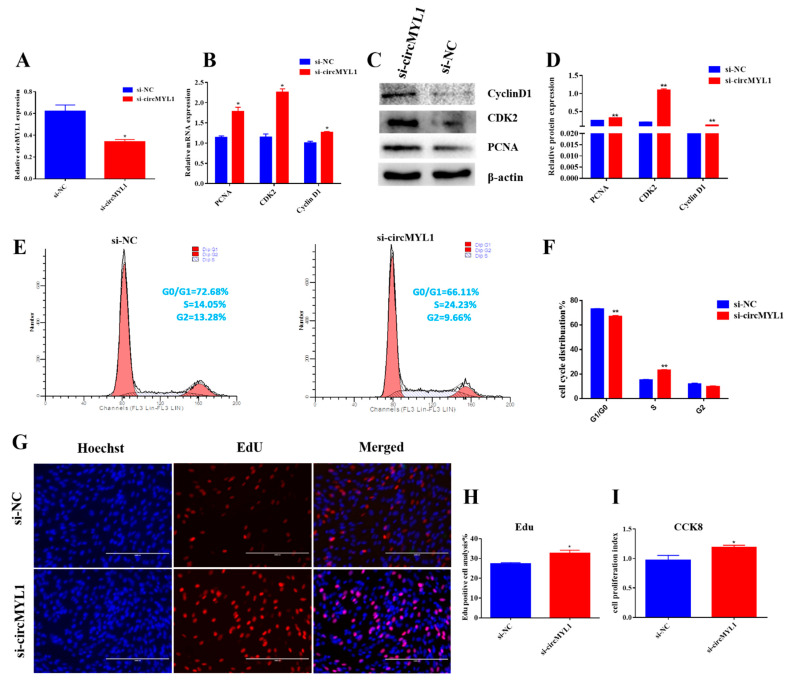
Knockdown of circMYL1 promotes the proliferation of bovine primary myoblasts (**A**) qRT-PCR detected the interference effect of siRNA (si-circMYL1). (**B**) qRT-PCR detected the relative expression levels of proliferation marker genes *PCNA*, *CDK2*, and *CyclinD1*. (**C**) Western blot detected the protein expression levels of proliferation marker genes *PCNA*, *CDK2*, and *CyclinD1*. (**D**) Histogram graph showing the protein band density evaluated using ImageJ. (**E**) Bovine primary myoblasts were transfected with si-circMYL1, and the cell cycle was analyzed by flow cytometry. (**F**) Histogram graph showing the number of cells in each phase of the cell cycle. (**G**) The cell proliferation was detected by (EdU) assay after transfection of si-circMYL1. EdU staining (red) for positive cells. DAPI staining (blue) for the cell nuclei. Scale bar, 200 μm. (**H**) Graph of a histogram showing the percentage of EdU-positive cells, and counted using ImageJ. (**I**) The cell counting kit-8 (CCK-8) assay was used to detect the cell proliferation index. Data are shown as mean ± SEM. *n* = 3. * *p* < 0.05; ** *p* < 0.01.

In order to explore the function of circMYL1 in the proliferation of skeletal muscle cells, we conducted overexpression and knocked down experiments by transfecting circMYL1 overexpression vector and small interfering RNAs (siRNAs) that was designed to target circMYL1 back-splicing (pCD2.1-circMYL1 and si-circMYL1) to bovine primary myoblasts. The relative expression of circMYL1 was detected by qRT-PCR after 48 h post-transfection, resulting in both the effect of overexpression and knockdown, reaching a substantial level (Figure 2A and Figure 3A). Furthermore, we detected the proliferation process of bovine primary myoblast by flow cytometry for cell cycle analysis, cell counting kit-8 (CCK-8), 5-ethynyl-2′-deoxyuridine (EdU) incorporation assays, RT-qPCR, and Western blotting assays after transfecting with pCD2.1-circMYL1/NTC, or si-circMYL1/si-NC. Firstly, we detected the effect of circMYL1on expression of the proliferation marker genes (*PCNA*, *CyclinD1*, and *CDK2*) using qRT-PCR and Western blotting and the results showed that the overexpression of circMYL1 significantly decreased the expression of these genes at the mRNA and protein levels (Figure 2B–D). Then, the cell cycle analysis revealed that overexpression of circMYL1 decreased the number of cells in the S phase and increased the number of cells in the G0/G1 phase (Figure 2E,F). The results were further confirmed by EdU staining. The results showed that the number of EdU-positive cells was less than that in the control group (Figure 2G,H). Furthermore, the CCK-8 assay revealed that circMYL1 possessed lower proliferation vitality than the negative control (Figure 2I). In addition, the bovine primary myoblasts were transfected with si-circMYL1, and we found that the knockdown of the circMYL1 increased the expression of the proliferation marker genes (*PCNA*, *CyclinD1*, and *CDK2*) at the mRNA and protein levels (Figure 3B–D). The cell cycle analysis revealed that si- circMYL1 increased the number of cells in the S phase and decreased the number of cells in G0/G1 (Figure 3E,F). Moreover, knockdown of circMYL1 markedly increased the number of EdU labelled cells (*p* < 0.05) compared with the negative control group (Figure 3G,H). Furthermore, the cell counting assay (CCK8) demonstrated that transfected with si-circMYL1 possessed higher proliferation vitality than the negative control (Figure 3I). These results revealed that circMYL1 inhibits the proliferation of bovine primary myoblasts.

### 3.3. circMYL1 Promotes Myoblasts Differentiation

**Figure 4:**
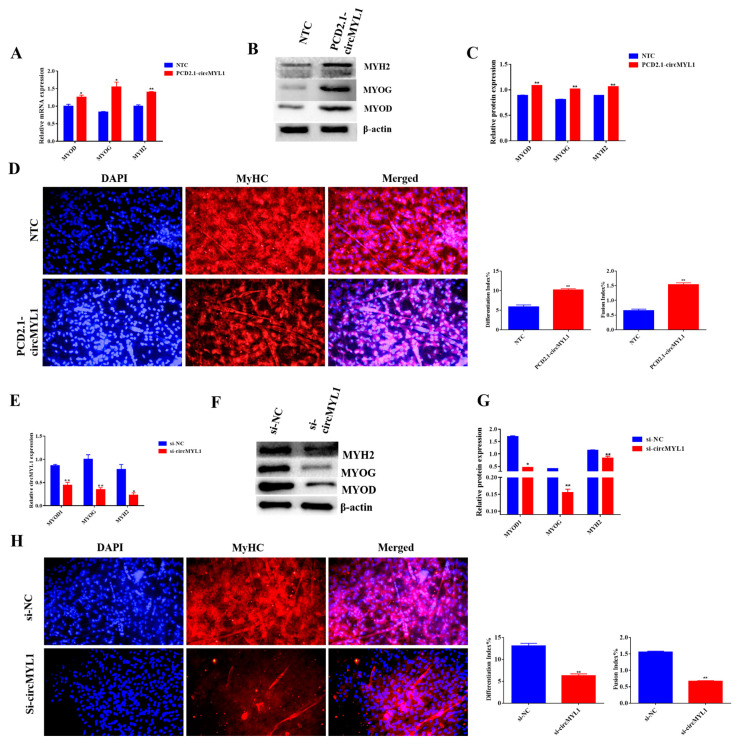
The effect of circMYL1 on the differentiation of myoblasts. The bovine primary myoblasts were transfected by pCD2.1-circMYL1/NTC or si-circMYL1/si-NC and differentiated cells for four days. Then, the cells were harvested. (**A**) qRT-PCR detected the relative expression levels of differentiation marker genes *MyoD*, *MyoG*, and *MYH2* after transfection with the circMYL1 overexpression vector. (**B**) Western blot detected the protein expression levels of differentiation marker genes *MyoD*, *MyoG*, and *MYH2*. (**C**) Histogram graph showing the protein band density evaluated using ImageJ. (**D**) Immunofluorescence detected MyHC (red)-positive myotubes after transfected by the circMYL1 overexpression vector, and the differentiation index and fusion index were counted. (**E**) qRT-PCR detected the relative expression levels of differentiation marker genes *MyoD*, *MyoG*, and *MYH2* after transfected with si-circMYL1. (**F**) Western blot detected the protein expression levels of differentiation marker genes *MyoD*, *MyoG*, and *MYH2*. (**G**) Histogram graph showing the protein band density evaluated using ImageJ. (**H**) Immunofluorescence detected MyHC (red)-positive myotubes after transfection by si-circMYL1. And they were detected under a fluorescence microscope: scale bar, 200 μm, and the differentiation index and fusion index were counted. Data are shown as mean ± SEM. *n* = 3. * *p* < 0.05; ** *p* < 0.01.

To investigate the possible roles of circMYL1 in the regulation of skeletal muscle differentiation, the bovine primary myoblasts were transfected by pCD2.1-circMYL1/NTC or si-circMYL1/si-NC and differentiated cells for four days. Then, the cells were harvested, and qRT-PCR and Western blotting analysis were conducted to detect the expression level of the differentiation marker genes (*MyoD*, *MyoG*, and *MYH2*). The result of qRT-PCR demonstrated that the overexpression of circMYL1 increased the expression levels of MyoD, MyoG, and MYH2 compared with the control group (Figure 4A). In contrast, the knockdown of circMYL1 decreased the expression level of MyoD, MyoG, and MYH2 mRNA compared with the si-NC group (Figure 4E).

Similarly, the Western blotting analysis showed that the overexpression of circMYL1 increased the protein levels of MyoD, MyoG, and MYH2 (*p* < 0.001) compared with the control group (Figure 4B,C), whereas, the knockdown of circMYL1 decreased the protein level of MyoD, MyoG, and MYH2 compared with the si-NC group (Figure 4F,G). Collectively, these results indicated that circMYL1 might promote bovine myoblast differentiation. Furthermore, the results from the immunofluorescence experiment found that cirMYL1 overexpression enhanced myotube formation. Meanwhile, the differentiation index and fusion index were both increased after differentiation (Figure 4D), whereas the knockdown of circMYL1 reduced the differentiation index and fusion index and the formation of myotube (Figure 4H).

### 3.4. circMYL1 Acts as A Sponge for miR-2400

**Figure 5:**
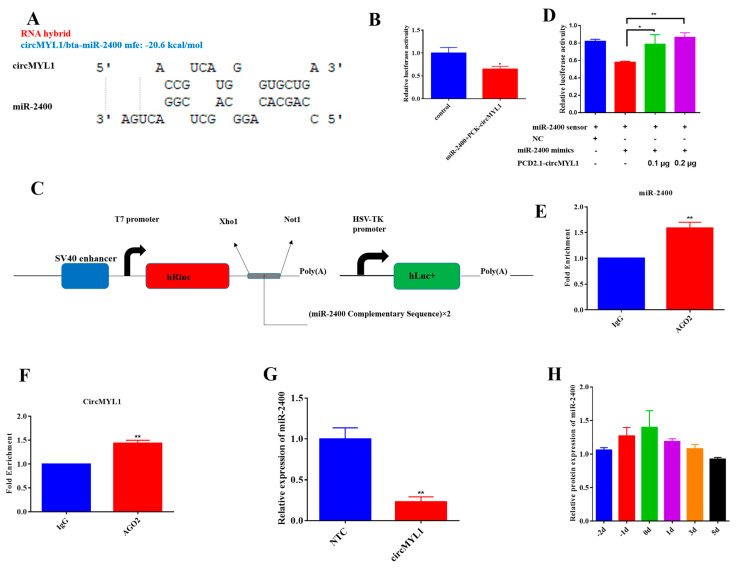
circMYL1 acts as a miR-2400 sponge (**A**) RNAhybrid predicted the potential binding sites of miR-2400 on the circMYL1 sequence. (**B**) Luciferase assay was conducted after co-transfection of the HEK293T cells with miR-2400 mimic and PCK-circMYL1. (**C**) A schematic drawing for the structure of the miR-2400 sensor. (**D**) Luciferase assay was conducted after co-transfected the miR-2400 sensor into HEK293 T cells with the miR-2400 mimic and/or PCD2.1-circMYL1. (**E**) AGO2 RNA immunoprecipitation (RIP) assay detected the fold enrichment of miR-2400 in bovine primary myoblasts. (**F**) AGO2 RNA immunoprecipitation (RIP) assay detected the fold enrichment of circMYL1 in bovine primary myoblasts. (**G**) qRT-PCR detected the relative expression levels of miR-2400 after overexpression of the circMYL1. (**H**) miR-2400 expression level during proliferation and differentiation of bovine primary myoblasts. Data are shown as mean ± SEM. *n* = 3. * *p* < 0.05; ** *p* < 0.01.

Previously, it has been found that circular RNA act as miRNA sponge and circMYL1 could inhibit proliferation and promote differentiation of bovine primary myoblasts. We conjectured that circMYL1exerts function by sponging miRNA and regulating miRNA expression. To screen the potential of miRNAs binding with circMYL1, a RNAhybrid assay was used to conduct the putative complementarity site between circMYL1 and miRNA. Remarkably, we found that circMYL1has potential miR-2400 binding site (Figure 5A). The dual-luciferase assay was used to explore whether circMYL1 target miR-2400 or not.

The result revealed that miR-2400 reduced the Rluc expression of psiCHECK-2-circMYL1 (PCK-circMYL1) in HEK293T cells (Figure 5B). Furthermore, we generated a sensor for miR-2400 by inserting two copies of the miR-2400 complementary sequence into the psiCHECK-2 vector (Figure 5C). The result showed that miR-2400 markedly reduced the Rluc activity of the miR-2400 sensor in HEK293T cells, on the other hand, the overexpression of the circMYL1 partially returned the decreased Rluc activity that made by binding miR-2400 in a dose-dependent manner (Figure 5D). Furthermore, RNA immunoprecipitation (RIP) assay was conducted in bovine primary myoblasts to confirm the interaction between circMYL1 and miR-2400, and qRT-PCR results showed positive enrichment of miR-2400 (Figure 5E) and circMYL1 (Figure 5F) in Argonaute 2 (Ago2) pull-down samples as compared to the negative control (IgG), indicating that circMYL1 binds to miR-2400 through the Ago2 protein. Moreover, the transfection with circMYL1 inhibits the relative expression level of miR-2400 in bovine primary myoblasts (Figure 5G). Additionally, we analyzed the expression level of miR-2400 during myogenesis in bovine primary myoblasts, and the result showed that the expression of miR-2400 was increased during the proliferation period and decreased during differentiation (Figure 5H). From these results, the expression levels of miR-2400 were opposite to circMYL1 expression during myogenesis. Altogether, these finding indicated that circMYL1 regulate proliferation and differentiation of bovine primary myoblast by sponging miR-2400.

### 3.5. miR-2400 Promotes Myoblast Proliferation

**Figure 6:**
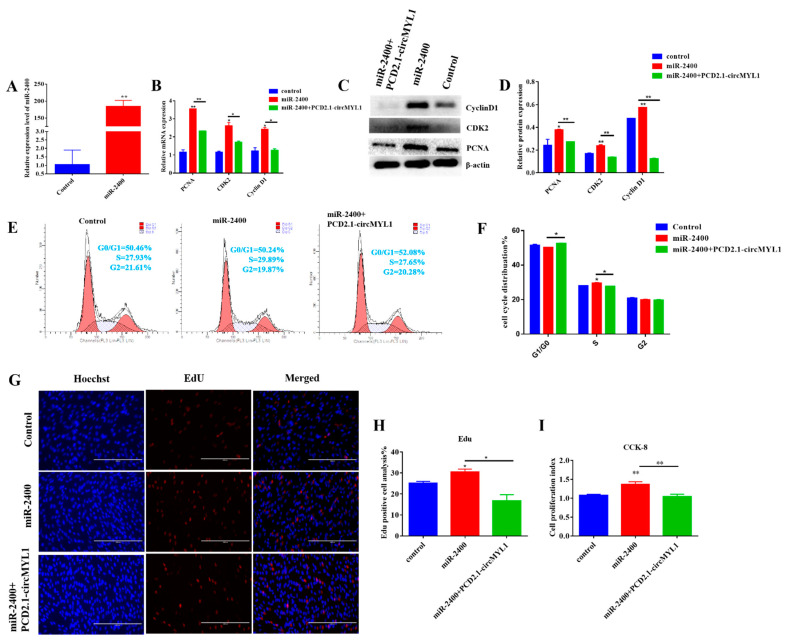
miR-2400 promotes myoblasts proliferation. (**A**) qRT-PCR detected the transfection efficiency of miR-2400 mimic. (**B**) Bovine primary myoblasts were transfected with miR-2400 mimic and/or circMYL1 overexpression vector, and qRT-PCR detected the relative expression levels of proliferation marker genes *PCNA*, *CDK2*, and *CyclinD1*. (**C**) Western blot detected the protein expression levels of proliferation marker genes *PCNA*, *CDK2*, and *CyclinD1*. (**D**) Histogram graph showing the protein band density evaluated using ImageJ. (**E**) The cell cycle was analyzed by flow cytometry. (**F**) Histogram graph showing the number of cells in each phase of the cell cycle. (**G**) The cell proliferation was detected by (EdU) assay Scale bar, 200 μm. (**H**) Graph of a histogram showing the percentage of EdU-positive cells. (**I**) The cell counting kit-8 (CCK-8) assay was used to detect the cell proliferation index. Data are shown as mean ± SEM. *n* = 3. * *p* < 0.05; ** *p* < 0.01.

To explore the role of miR-2400 during myoblast proliferation, the bovine primary myoblasts at 40% density were transfected, by 50 nm miR-2400 mimic and 50 nm mimic negative control and pCD2.1-circMYL1/NTC for 48 h. The expression of miR-2400 was increased by mimic compared with mimic control (Figure 6A). Various methods, such as qRT-PCR, Western blotting, cell counting kit-8 assay, and EdU, were used to detect the potential role of miR-2400 in the proliferation of myoblast. The qRT-PCR results demonstrated that the transfected by miR-2400 mimic increased the relative expression levels of proliferation marker genes (*PCNA*, *CyclinD1*, and *CDK2*) at mRNA level, but this result was abolished when transfected by overexpression of circMYL1 (Figure 6B). Similarly, the Western blotting analysis showed that the miR-2400 overexpression increased the protein expression level of (*PCNA*, *CyclinD1*, and *CDK2*) genes, and also, this result was reversed by transfected by circMYL1 overexpression (Figure 6C,D). Analysis of the cell cycle showed that the miR-2400 mimic increased the number of cells in the S phase and decreased the proportion of cells in the G0/G1 phase, indicating that miR-2400 may promote the proliferation of bovine myoblast (Figure 6E,F). Moreover, 5-ethynyl-2′-deoxyuridine (EdU) analysis and cell counting assay (CCK-8) and showed that overexpression of miR-2400 significantly up-regulated the cell proliferation (Figure 6G–I). However, the result showed that when we co-transfected the miR-2400 mimic and PCD2.1-circMYL1 into bovine primary myoblasts, the overexpression of circMYL1 eliminated the effect of miR-2400 on the proliferation of myoblasts (Figure 6D–F).

### 3.6. miR-2400 Inhibits Myoblast Differentiation

**Figure 7:**
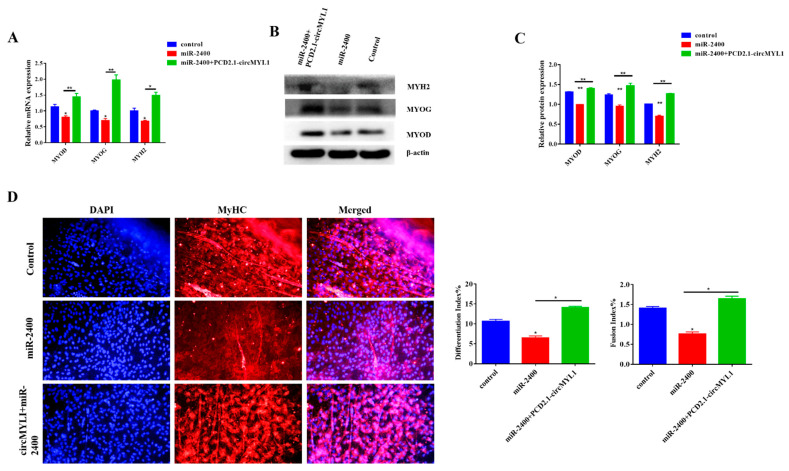
The effect of miR-2400 on the differentiation of myoblasts. (**A**) Bovine primary myoblasts were transfected with miR-2400 mimic and/or circMYL1 overexpression vector, and qRT-PCR detected the relative expression levels of differentiation marker genes *MyoD*, *MyoG*, and *MYH2*. (**B**) Western blot detected the protein expression levels of differentiation marker genes *MyoD*, *MyoG*, and *MYH2*. (**C**) Histogram graph showing the protein band density evaluated using ImageJ. (**D**) Immunofluorescence detected MyHC (red)-positive myotubes after transfected the bovine primary myoblasts with miR-2400 mimic and/or circMYL1 overexpression vector, and they were detected under a fluorescence microscope: scale bar, 200 μm, and the differentiation index and fusion index were counted. Data are shown as mean ± SEM. *n* = 3. * *p* < 0.05; ** *p* < 0.01.

Potential roles of miR-2400 in the regulation of skeletal muscle differentiation were analyzed through transfecting bovine primary myoblasts with miR-2400 mimic or PCD2.1-circMYL1 at 70%–80% density and differentiated cells for four days. The cells were collected, qRT-PCR and Western blotting analysis were conducted for the expression analysis of the differentiation marker genes (*MyoD*, *MyoG*, and *MYH2*). The result of qRT-PCR demonstrated that the miR-2400 mimic decreased the expression levels of MyoD, MyoG, and MYH2 at mRNA compared with the control group, in contrast, when co-transfected with PCD2.1-circMYL1 and miR-2400, the expression levels of MyoD, MyoG, and MYH2 at mRNA were increased (Figure 7A). Furthermore, the Western blotting analysis showed that the miR-2400 mimic decreased the protein levels of MyoD, MyoG, and MYH2 compared with the control group, whereas, co-transfected with PCD2.1-circMYL1 and miR-2400, increased the protein level of MyoD, MyoG, and MYH2 (Figure 7B,C). Immunofluorescence assay showed that miR-2400 reduced the differentiation index and fusion index and myotube formation, but circMYL1 overexpression reversed this effect to some extent (Figure 7D). Collectively, these results confirmed that miR-2400 inhibits the differentiation of bovine primary myoblasts; however, these effects can be eliminated by overexpression of circMYL1. In addition, these findings also show that circMYL1 promotes myogenesis by sponging miR-2400.

### 3.7. circMYL1 Acts as a ceRNA for miR-2400 to Activate MYOG

**Figure 8:**
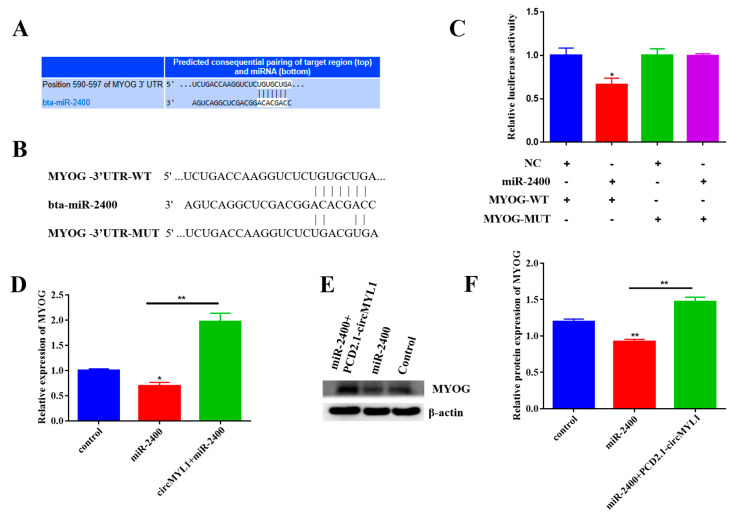
circMYL1 acts as a ceRNA for miR-2400 to activate MYOG (**A**) TargetScan 7.2 predicted the potential binding site of miR-2400 in the MYOG-3′-UTR. (**B**) The sequence of miR-2400 and its predicted binding site in the MYOG 3′-UTR and mutated 3′-UTR. (**C**) Luciferase assay was conducted after co-transfected the miR-2400 mimic or negative control into HEK293 T cells with the MYOG-WT or MYOG-MUT. The activity of renilla luciferase was normalized for the activity of firefly luciferase. (**D**) qRT-PCR detected the relative expression levels of MYOG in bovine primary myoblasts after transfected with miR-2400 mimic and/or circMYL1 overexpression vector. (**E**) Western blot analysis detected the protein expression levels of MYOG in bovine primary myoblasts after transfected with miR-2400 mimic and/or circMYL1 overexpression vector. (**F**) Histogram graph showing the protein band density evaluated using ImageJ. Data are shown as mean ± SEM. *n* = 3. * *p* < 0.05; ** *p* < 0.01.

**Figure 9:**
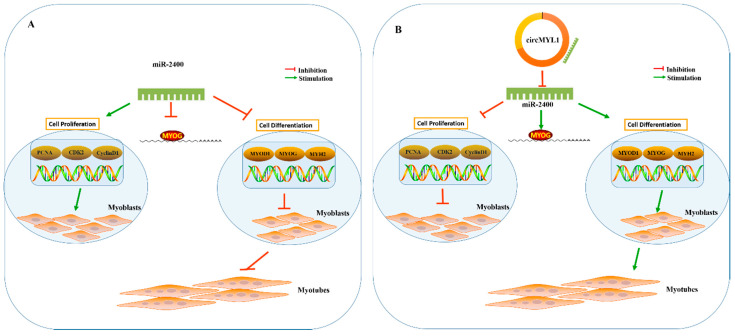
Schematic mechanism of miR-2400 and circMYL1 in regulating bovine myogenesis. (**A**) MiR-2400 regulates myogenesis by targeting MYOG. MiR2400 promotes the proliferation of bovine primary myoblasts through the up-regulation of proliferation marker genes *PCNA*, *CDK2*, and *CyclinD1*. MiR-2400 inhibits the differentiation of bovine primary myoblasts through the negative regulation of differentiation marker genes *MyoD*, *MyoG*, and *MYH2*. (**B**) CircMYL1 regulates myogenesis by acting as a ceRNA for miR-2400 to activate MYOG. CircMYL1 inhibits the proliferation of bovine primary myoblasts through negative regulation of proliferation marker genes *PCNA*, *CDK2*, and *CyclinD1*. CircMYL1 promotes the differentiation of bovine primary myoblasts through the up-regulation of differentiation marker genes *MyoD*, *MyoG*, and *MYH2*.

The online bioinformatics suite TargetScan 7.2 was used to clarify miR-2400′s possible molecular regulatory mechanism for inhibiting proliferation and promoting differentiation of bovine primary myoblasts. MYOG was considered as a potential target gene for miR-2400 (Figure 8A). We then constructed MYOG-3′-UTR (wild (WT) and mutant (MUT)) luciferase reporter vectors that contain potential miR-2400 binding sites (Figure 8B). The two plasmids were transfected separately into HEK293T cells with miR-2400 mimic or negative control. MYOG-WT’s luciferase activity was significantly (*p* < 0.05) decreased by miR-2400 but did not affect MYOG-MUT (Figure 8C). To confirm the involvement of circMYL1 in the miR-2400 mediated the regulation of MyoG expression, bovine primary myoblast was transfected by pCD2.1-circMYL1 and/or miR-2400 mimic, and the result showed that the overexpression of miR-2400 decreased MyoG expression at mRNA and protein level (Figure 8D–F); however, this effect was reversed when transfected with circMYL1. All these results, suggesting that circMYL1 regulates the proliferation and differentiation of bovine myoblasts by acting as a miR-2400 sponge to activate the MYOG gene (Figure 9).

## 4. Discussion

Recently, a large number of circular RNAs have been identified in different cells and tissues as the results of the development of high-throughput sequencing [15,36]. cirRNAs play a significant biological role in many physiological and pathological processes as a new post-transcription regulator and are a hot topic in biological research [37]. However, the regulatory role of circRNAs in the growth and development of skeletal muscle is still unclear. Myosin light chain 1 (*MYL1*) is a well-known gene, particularly related to calcium regulation, and plays a vital role in muscle growth and differentiation [38]. It was found that *MYL1* is a critical gene for skeletal muscle function and the deficiency of *MYL1* related to the severe congenital myopathy [39]. The circMYL1 derived from exon four and five of the *MYL1* gene. circRNAs are more stable and have a longer half-life compared to linear mRNAs because of its covalently closed circular structure [14]. Recently, several studies have proven the crucial roles of circular RNAs in the proliferation and differentiation of myoblasts. There confirmed the role of circFUT10 [28], circSVIL [8], circFGFR4 [25], circFGFR2 [40], and circSNX29 [29] in the enhance of myogenesis through positively regulated myoblast differentiation. Moreover, it was found that circLMO7 [27], circZNF609 [26], negatively regulated myoblast differentiation.

In our previous publication, the whole-genome sequencing data explored the highest expression level of circMYL1 (FPKM = 1869.8) between differentially expressed circular RNAs (up-regulated), and differentially expressed between adult and fetal of bovine muscle tissues suggesting that circMYL1 has an essential role in muscle development [27]. In the current study, we investigated the possible role of circMYL1 in muscle development. The expression of circMYL1 in different cattle tissues was highly expressed in skeletal muscle. Additionally, the expression decreased during the proliferation and increased during primary myoblast differentiation. These results suggesting that the circMYL1 may inhibit proliferation promote differentiation of skeletal muscle. In our previous research, we confirmed that circFUT10 and circSNX29 inhibit proliferation and promote differentiation of bovine primary myoblasts [28,29]. To assess this hypothesis, firstly, we studied the function of circMYL1 in bovine primary myoblast proliferation. The results showed that the mRNA and protein expression levels of proliferation marker genes such as *PCNA*, *CyclinD1*, and *CDK2* were decreased in circMYL1-overexpressed cells, whereas the opposite effects were detected in circMYL1-knockdown cells. Moreover, the EdU incorporation analysis exposed that EdU-positive cells were reduced by overexpression of circMYL1 and increased by knockdown of circMYL1. Furthermore, the cell counting assay (CCK8) and cell cycle analysis showed that circMYL1 reduced the proliferation of myoblasts. Based on these results, we strongly postulated that circMYL1 has a vital role in myoblast cell proliferation. Furthermore, we also found that the overexpression of circMYL1 increased the expression levels of differentiation marker genes (*MyoD*, *MyoG*, and *MYH2*); however, the expression levels decreased by knockdown of circMYL1. The protein levels of MyoD, MyoG, and MYH2 showed similar expression. Myosin heavy chain immunofluorescence also confirmed that circMYL1 promoted the differentiation of bovine primary myoblasts into myotubes. These results further confirming the vital function of circMYL1 in the skeletal muscle cell, and it can promote myoblast differentiation.

Previously, it was investigated that circRNAs can act as competing for endogenous RNAs, which can sponge miRNAs to regulate the expression of post-transcription genes. In our previous studies we reported that some circRNAs regulated the development of bovine skeletal muscles by sponging various miRNAs, circTTN acts as a miR-432 sponge to promote myoblast proliferation and differentiation through the IGF2/PI3K/AKT signalling pathway [41], circSNX29 inhibit cell proliferation and promote cell differentiation by sponging miR-744 to activate the Wnt5a/ca^2+^ pathway [29], circLMO7 sponge miR-378a-3p to inhibit cell differentiation and promote cell proliferation of myoblasts [27], and circFGFR4 promote cell differentiation by sponging miR-107 to increase the expression of Wnt3a [25]. In this research, we used bioinformatics software to predict the potential targeting miRNAs, RNAhybrid assay showed that circMYL1 have possible biding site for miR-2400. Furthermore, we validated that the miR-2400 as targeting for circMYL1 using the dual-luciferase reporter assay, RNA immunoprecipitation, qRT-PCR, and Western blotting analysis. The results suggested that circMYL1 inhibits proliferation and promotes differentiation of myoblast by sponging miR-2400. It was reported that miR-2400 is a novel and specific bovine miRNA because it is not detectable in other mammalian genomes [33]. It was reported that in skeletal muscle-derived satellite cells (MDSCs), the overexpression of miR-2400 promotes proliferation by targeting the *MYOG* gene [34]. In this study, we also find the same function of miR-2400 in bovine primary myoblast proliferation. Furthermore, we evaluated the vital role of miR-2400 in myoblast differentiation; miR-2400 inhibits the bovine primary myoblast differentiation, which was never done before. The findings were suggesting that miR-2400 acts as a negative regulatory factor during myoblasts differentiation. Conversely, during muscle development, the roles of miR-2400 were opposite to the effects of circMYL1. Therefore, circMYL1 may act as a molecular sponge for miR-2400. Furthermore, we found that circMYL1 inhibited the stimulatory effects of miR-2400 on the proliferation and eliminated the inhibitory effects of miR-2400 in the differentiation of bovine primary myoblast.

## 5. Conclusions

In the present study, we identified the novel circular RNA of circMYL1, produced by the *MYL1* gene that could regulate the proliferation and differentiation of bovine myoblasts by acting as a miR-2400 sponge to activate the *MYOG* gene. The findings show a new mechanism involved in muscle development and could be helpful for cattle breeding programs.

## Data Availability

The data used to support the findings of this study are available from the corresponding author upon request.

## Supplementary Materials

The following are available online at https://www.mdpi.com/2073-4409/10/1/176/s1, Table S1. Primers for qPCR, Table S2. Primers for vector construction, Text S1. Sequence of cattle circMYL1.
