# Supplementary material for: Circular RNA circMYL1 Inhibit Proliferation and Promote Differentiation of Myoblasts by Sponging miR-2400
Source: Cells. 2021 Jan 16;10(1):176. doi: 10.3390/cells10010176 (PMC7830797; doi:10.3390/cells10010176)
Supplement: Supplementary file 1 [file cells-10-00176-s001.pdf]

Supplemental Information

Table S1. Primers for qPCR

Name	Forward primer 5’→3’	Reverse primer 5’→3’
CircMYL1	AAGAAGAAGTGGAAGCGCTGA	TGCCCTGGTCCTTGTTGTTGG
Bta-miR-2400	TCGCCAGCACAGGCAGCTCG	GCAGGGTCCGAGGTATTC
Bta-miR-2400-RT	GTCGTATCCAGTGCAGGGTCCGAGGTATTCGCACTGGATACGACTCAGTC	
U6	GGAAGAGGGCCTATTTCCCAT	CCCAACTTCTCGGGGACTGTG
GAPDH	TGAGGACCAGGTTGTCTCCTGCG	CACCACCCTGTTGCTGTAGCCA
MYOD	CCCAAAGATTGCGCTTAAGTG	GTTCCTTCGCCTCTCCTACCT
MYOG	AGGGGATCATCTGCTCCCAG	ATCCCGGCAGACAATCTCAG
MYH2	CTGAATCCCAGGTCAACAAG	TCATCCCATAGCATCAGGAC
PCNA	GTCAGGAGTCAACCAAGAAAG	CTGTCAGTCTCCTTCCCTAAC
CDK2	TTTGCTGAGATGGTGACCCG	TAACTCCTGGCCAAACCACC
CyclinD1	GACGAGCTGCTGCACATGGA	TGCTTGTTCTCCTCGGCCAC
MYL1	TGTCCAAATCTGTTTACCATC	ACTTAGTTTGTGTGGTATGAA
CircMYL1. convergent primer	TGCGTGTCTTCGACAAGGAA	TTTCATCTTTTCACCTAGTGTGGC

Table S2. Primers for vector construction

Name	Primer sequence 5' →3'
PCD2.1-circMYL1-F	CGGGATCCCTTTGTTGAGGGTCTGCGTGT
PCD2.1-circMYL1-R	GGGGTACCTCTTCATAAGTGCCCTGGTCC
psiCHECK2-circMYL1-F	CCGCTCGAGCTTTGTTGAGGGTCTGCGTGT
psiCHECK2-circMYL1-R	ATAAGAATGCGGCCGCTCTTCATAAGTGCCCTGGTCC
psiCHECK2-miR-2400-Sensor-F	TCGATCAGTCCGAGCTGCCTGTGCTGGTCAGTCCGAGCTGCCTGTGCTGG
psiCHECK2-miR-2400-Sensor-R	GGCCCCAGCACAGGCAGCTCGGACTGACCAGCACAGGCAGCTCGGACTGA
psiCHECK2-MYOG-W-F	CCGCTCGAGGCCTGCCCTGAATTGAGATGA
psiCHECK2-MYOG-W-R	ATAAGAATGCGGCCGCATCCCTGGCAACTTCAGCAC
psiCHECK2-MYOG-MUT -F	CCGCTCGAGATCTGACCAAGGTCTCTGACGTGAAGTTGC
psiCHECK2-MYOG-MUT -R	ATAAGAATGCGGCCGCCTAGCACCCAGTCTTTATTT

Text S1. Sequence of cattle circMYL1

>circRNA2388

AGATGAATGCCAAGAAAATTGAGTTTGAACAATTTCTGCCCATGTTGCAAGCTATTT
CCAACAACAAGGACCAGGGCACTTATGAAGACTTTGTTGAGGGTCTGCGTGTCTTCG
ACAAGGAAGGCAATGGCACCGTCATGGGTGCTGAACTTCGTCATGTTCTAGCCACAC
TAGGTGAAAAGATGAAAGAAGAAGTGGAAGCGCTGATGGCAGGTCAGGAAGA
CTCCAATGGCTGCATCAACTATGAAG
